# Epigenetic regulation of innate immune memory in microglia

**DOI:** 10.1186/s12974-022-02463-5

**Published:** 2022-05-14

**Authors:** Xiaoming Zhang, Laura Kracht, Antonio M. Lerario, Marissa L. Dubbelaar, Nieske Brouwer, Evelyn M. Wesseling, Erik W. G. M. Boddeke, Bart J. L. Eggen, Susanne M. Kooistra

**Affiliations:** 1 Department of Biomedical Sciences of Cells and Systems, Section Molecular Neurobiology, University Medical Center Groningen, University of Groningen, Antonius Deusinglaan 1, Hpc-FB43, 9713 AV Groningen, The Netherlands; 2 Department of Internal Medicine, Division of Metabolism, Endocrinology, and Diabetes, University of Michigan, Ann Arbor, MI USA; 3 Center for Healthy Aging, Department of Cellular and Molecular Medicine, University of Copenhagen, Blegdamsvej 3B, 2200 Copenhagen, Denmark

**Keywords:** Microglia, Chromatin, Tolerance, Priming, Innate immunity, Neuroinflammation

## Abstract

**Background:**

Microglia are the tissue-resident macrophages of the CNS. They originate in the yolk sac, colonize the CNS during embryonic development and form a self-sustaining population with limited turnover. A consequence of their relative slow turnover is that microglia can serve as a long-term memory for inflammatory or neurodegenerative events.

**Methods:**

Using ATAC-, ChIP- and RNA-sequencing, we characterized the epigenomes and transcriptomes of FACS-purified microglia from mice exposed to different stimuli. A repeated endotoxin challenge (LPS) was used to induce tolerance in microglia, while genotoxic stress (DNA repair deficiency-induced accelerated aging through Ercc1 deficiency) resulted in primed (hypersensitive) microglia.

**Results:**

Whereas the enrichment of permissive epigenetic marks at enhancer regions could explain training (hyper-responsiveness) of primed microglia to an LPS challenge, the tolerized response of microglia seems to be regulated by loss of permissive epigenetic marks. We identify that inflammatory stimuli and accelerated aging as a result of genotoxic stress activate distinct gene networks. These gene networks and associated biological processes are partially overlapping, which is likely driven by specific transcription factor networks, resulting in altered epigenetic signatures and distinct functional (desensitized vs. primed) microglia phenotypes.

**Conclusion:**

This study provides insight into epigenetic profiles and transcription factor networks associated with transcriptional signatures of tolerized and trained microglia in vivo, leading to a better understanding of innate immune memory of microglia.

**Supplementary Information:**

The online version contains supplementary material available at 10.1186/s12974-022-02463-5.

## Background

Microglia are of myeloid lineage and are long-lived tissue-resident macrophages of the central nervous system (CNS) parenchyma [1].

Macrophages possess innate immune memory (IIM). IIM describes the concept that macrophages, after experiencing a primary ‘priming’ or ‘desensitizing’ stimulus, react with a stronger (immune training) or weaker (immune tolerance) immune response to a subsequent stimulus [2, 3]. IIM was discovered and has been extensively described in blood-derived monocytes/macrophages [4–8].

Similar functional states have been described for microglia in mouse models [2, 9]. Primed microglia can be elicited in mouse models of prion disease [10], neurodegeneration [11, 12], natural aging [13] and neuronal genotoxic stress-induced accelerated aging [14]. When these mice experienced a peripheral lipopolysaccharide (LPS) challenge, microglia exhibited an excessive immune response manifested by increased expression of pro-inflammatory cytokines, called microglia training [10, 11, 13, 14]. Oppositely, mouse microglia can be desensitized with LPS [15–17]. After a secondary challenge, in the form of LPS [16, 17], traumatic brain injury [15] or cerebral ischemia [18], microglia display immune tolerance which is defined by a reduced immune response. Interestingly, In vitro studies with primary microglia suggest that the dosage and timing of pathogen exposure is decisive for the emergence of immune training or tolerance ([19, 20]). Whether this is also the case in vivo, remains to be determined. 

Microglia are implicated in CNS development, and neurodevelopmental and neurodegenerative diseases [21–27]. It is especially interesting to investigate microglia IIM in this context. The combination of perturbations like maternal immune activation during vulnerable periods of CNS development together with the occurrence of multiple stimuli over a long period of time is thought to cause neurodevelopmental or neurodegenerative diseases. Microglia are the prime cells that respond to CNS stimuli since they express a wide range of cell surface receptors and adhesion molecules (homeostatic gene signature) through which they can sense those endogenous and exogenous stimuli [28–34].

Epidemiologic studies report that infections during specific periods of pregnancy increase the risk for the child to develop neurodevelopmental disorders, like autism or schizophrenia [35]. Mouse models of maternal immune activation suggest a role for microglia IIM in this process [2, 36]. Peripheral LPS challenge of pregnant mouse dams caused preconditioning of offspring microglia which long-lastingly affects microglia LPS responsiveness in adult offspring and also caused behavioral abnormalities [16].

In case of neurodegenerative diseases, genetic risk loci are generally immune-related [37, 38] and specifically enriched in microglia [39]. A common gene signature was identified in multiple mouse models of neurodegenerative diseases, aging and priming and encompasses genes, such as *Axl*, *Clec7a* and *Mac2* [27]. This microglia transcriptional phenotype is orchestrated by the APOE–TREM2 pathway and is associated with altered phagocytic and lysosomal activity, and lipid metabolism [27, 40–42]. Given the chronic nature of neurodegenerative diseases, it is hypothesized that microglia are trapped in a primed/trained state ultimately leading to neurotoxicity [43, 44]. This hypothesis was recently confirmed by the observation that induction of priming of microglia in early adulthood caused exacerbation of Aβ pathology later in life, whereas desensitization of microglia diminished Aβ pathology in a mouse model of AD [11]. Current studies suggest microglia priming and tolerance to have neurotoxic [11, 16] or neuroprotective [11, 15, 18] consequences, respectively. However, these outcomes should not be generalized and the effects of microglia tolerance and priming on neuronal viability need to be elucidated in a context-specific manner [2].

Both tolerant and trained immunity of peripheral macrophages are long-lasting changes in functionality that are instructed by epigenetic reprogramming [6, 8, 45–47]. Though epigenetic programming has been clearly implicated in the segregation of microglia from other tissue-resident macrophages in both mouse and human [31, 32, 34], little is known about the changes in epigenetic signatures in microglia in response to (systemic) immune stimuli or endogenous neuronal damage and how epigenetic memory serves to change subsequent responses. Since microglia are relatively long-lived cells [48, 49], experience of past stimuli is long-lastingly secured in the microglial epigenome and can thus have persistent consequences on microglia functionality and neuronal viability. Several lines of evidence suggest a role for epigenetic regulation of microglia functional states [11, 17, 22, 41, 50–52].

To delineate the gene networks and associated epigenetic signatures and transcription factors that underlie functional microglia states of priming and tolerance, we acutely isolated microglia from mice challenged with LPS and from accelerated aging mice and analyzed their transcriptional and chromatin status at a genome-wide level.

## Methods

### Animals

Animals were conventionally housed in macrolon cages with open top under a 12/12 h light/dark cycle (8 p.m. lights off, 8 a.m. lights on) with ad libitum access to food and water. Climate in the animal facility was controlled and temperature set at 21 °C.

#### Tolerance induction

Male C57BL/6J mice were obtained at the age of 7–9 weeks with weights in the range of 25–30 g (Envigo, Horst, The Netherlands). Upon arrival, a minimum acclimatization time of 2 weeks was ensured, where mice were monitored weekly in terms of general appearance and weight. All animals were housed individually to prevent fighting induced wounds and inflammation and randomly assigned to experimental conditions.

To induce endotoxin tolerance, mice received 1 mg/kg body weight LPS (Sigma-Aldrich, *E. coli* 0111:B4, L4391) diluted in dPBS (Lonza, BE17512F) to a total volume of 200 µL by intraperitoneal injection. Immediately following LPS administration, mice were housed in a recovery cabinet at 26 °C for 24 h. The weight and general health of injected animals were monitored daily until the body weight was completely restored (usually within 7 days), and monitoring was continued after recovery at a weekly basis. All control mice received 200 µL dPBS by intraperitoneal injection. After 4 weeks, the mice received a second injection with either dPBS or LPS (1 mg/kg body weight, diluted in 200 µL dPBS).

#### Obtaining primed microglia

*Ercc1* transgenic mice [53] were bred in house by crossing *Ercc1*^wt/*292^ mice (FVB background, the *292 allele is hereafter indicated with Δ) with *Ercc1*^*wt/ko*^ mice (BL6 background) as previously described [14]. The offspring were genotyped after weaning using the primers listed in Table 1. *Ercc1*^*Δ/ko*^ were used as experimental mice while littermates with *Ercc1*^*wt/Δ*^, *Ercc1*^*wt/ko*^ or *Ercc1*^*wt/wt*^ genotypes were used as control. All the mice were group-housed in conventional cages. Initially, mice were monitored weekly, which increased to twice per week after the aging-related symptoms appeared. Accelerated aging in Ercc1 animals was monitored by gradual weight loss during aging and the occurrence of motor abnormalities, including clasping of the hind-limbs when lifted by the tail. Bottles with long drinking spouts were provided to prevent dehydration of *Ercc1*^*Δ/ko*^ animals. At 11–12 weeks of age, the mice received 1 mg/kg body weight LPS or dPBS as described above. Immediately following LPS administration, mice were temporarily housed in a recovery cabinet at 26 °C.

**Table 1:** *Ercc1* genotyping primers

Allele	Product size	Forward primer 5′–3′	Reverse primer 5′–3′
*WT*	246 bp	AGCCGACCTCCTTATGGAAA	ACAGATGCTGAGGGCAGACT
*KO*	390 bp	TCGCCTTCTTGACGAGTTCT	ACAGATGCTGAGGGCAGACT
292* (Δ)	530 bp	TCGCCTTCTTGACGAGTTCT	CTAGGTGGCAGCAGGTCATC

All animals were killed under deep anesthesia (4% isoflurane with 7.5% O_2_) and perfused with cold dPBS exactly 3 h after the last injection.

### Microglia isolation and flow cytometry

Microglia were isolated as previously described [54]. After perfusion, brains were removed from the skull and kept in cold medium A (HBSS (Gibco, 14170-088) with 0.6% glucose (Sigma, G8769) and 7.5 mM HEPES (Lonza, BE17-737E)). All subsequent steps were performed on ice, centrifugation was at 4 °C. Brains were dissociated using a Potter–Elvehjem tissue homogenizer after which the homogenate was passed over a 70 µM cell strainer (Corning, 352350) and pelleted by centrifugation at 220×*g* for 10 min. Next, myelin was removed by resuspending the pellet in 25 mL 24% Percoll (Fisher, 17-0891-01) in medium A (1 × final concentration) with 3 mL PBS layered on top, followed by centrifugation for 20 min at 950×*g* (acceleration 4 and brake 0). The microglia enriched cell pellets were incubated with CD11b-PE (clone M1/70, eBiosciences, 12-0112-82), CD45-FITC (clone 30-F11, eBiosciences, 11-0451-82), and Ly6c-APC (clone HK1.4, Biolegend, 128016) antibodies for 30 min on ice. Then the cells were washed once in medium A without phenol red and filtered into FACS tubes. Microglia were sorted by gating the DAPI^neg^CD11b^high^CD45^int^Ly6c^neg^ cells using the Beckman Coulter MoFlo Astrios or XDP. Microglia were collected in siliconized Eppendorf tubes (Sigma, T3406-250EA) containing medium A. Flow cytometry data were analyzed using FlowJo Analysis Software.

### RNA isolation and RNA sequencing

Total RNA was isolated using a Qiagen RNeasy Micro Kit (Qiagen, 74004) according to the manufacturer’s instructions. For every sample, we sorted 50.000 microglia into a separate tube for qPCR validation of an inflammatory response and collected the remaining microglia (usually 200.000–300.000 microglia per brain from wt animals and 150.000–200.000 for Ercc1Δ/ko animals) into another tube for RNA-seq. Quantification of the RNA showed the concentration of isolated RNA for sequencing was on average 3 ng/ul. 

#### Endotoxin tolerance

The quality of the total RNA was determined using an Experion (Biorad). All included samples had an RNA quality indicator > 6. Sequencing libraries were generated with a TruSeq RNA library prep kit (Illumina, RS-122-2001). Pooled libraries were sequenced with a HiSeq Rapid SBS kit (50 cycles, Illumina, FC-402-4022) using single reads on a HiSeq 2500 (Illumina).

#### Priming/Ercc1 knockout

The quality of total RNA samples isolated from *Ercc1*^*Δ/ko*^ mice was determined on a LabchipGX (PerkinElmer). All included samples had an RNA quality score > 5. Sequencing libraries were generated using NEXTflex® Rapid Illumina Directional RNA-Seq Library Prep Kit (BiooScientific, NOVA-5138-10) with polyA selection. Pooled libraries were sequenced using the NextSeq 500/550 High Output v2 kit (75 cycles, Illumina, FC-404-2005) with single reads on a NextSeq500 (Illumina).

### RNA-sequencing analysis

Samples (n = 3 per condition) were processed using our *in-house* pipeline, where quality control was performed with FastQC (v0.11.8). Adapter sequences were removed with bbduk (v38.69). The Ensembl genome *Mus musculus* (GRCm38.82) was used for alignment (STAR v2.7.3a, [55]). Sorting of the aligned reads was done with bamsort tool from biobambam2 tools (v2.0.95). featureCounts (v2.0.0, [56]) was used to quantify the reads. Picard (v1.130, [57]) and samtools were used to perform the quality control check and the generation of the fastq files. Downstream analyses were performed using R/Bioconductor packages (v.3.11), as briefly summarized. Specific functions from EdgeR (v3.30.3, [58]) were used for data normalization and calculation of rpkm expression values. Log2-CPM values and mean–variance relationship were calculated with the voom WithQualityWeights function from limma (v3.44.3, [59]). Unwanted/hidden sources of variation were removed using sva (v3.36.0, [60]). Differential expression analysis was performed using limma. Annotation was performed with biomaRt [61]. For plotting purposes, genes with a logFC > 1 and FDR < 0.01 were considered differentially expressed.

Clustering analysis was performed using the ward.D2 clustering method and Manhattan distance as clustering metrics. Heat maps were assembled using the pheatmap package (v1.0.12, [62]). The optimal number of gene clusters was estimated upon visual inspection of the heat maps. Gene ontology enrichment analysis of the gene clusters was performed with the ‘enrichGO’ function of the clusterProfiler package (v3.16.1, [63]). PCA plots, scatterplots and dotplots were made with the package ggplot2 (v3.3.2, [64]) and standard plot functions from R.

To visualize the overlap of differentially expressed genes of different comparisons (Additional file 2: Fig. S2E), gene lists of the indicated comparisons were ranked based on expression level. Following, percentiles were assigned to the ranked genes and Δpercentiles were calculated by subtracting percentiles of each gene from the two indicated comparisons. The results were depicted in a volcano plot, where the dots are differential expressed genes (logF > 1, FDR < 0.01) in the indicated comparison and the color of the dots shows overlap of gene expression levels from the two indicated comparisons.

For the Venn diagram in Fig. 5a, only upregulated genes of the PL versus PP, KO-PBS versus WT-PBS and KO-LPs versus WT-LPS differential gene lists (Additional files 5, 6) and genes of cluster 2 and 4 of the tolerance moue model (Additional file 7) were use. The Venn diagram was made with the ‘venn’ function of the gplots package (v3.1.0 [65]).

### ChIP-sequencing

The procedure of chromatin immunoprecipitation has been described previously [17]. Sorted microglia were fixed in 1 mL 1% formaldehyde diluted in dPBS at 20 °C for 10 min and fixation was stopped by adding glycine to a final concentration of 0.125 M glycine. Fixed cells were washed twice by 1 mL cold dPBS, and then lysed with cell lysis buffer (5 mM Pipes, pH 8.0; 85 mM KCl; 0.5% NP-40) by incubating on ice for 10 min. At the end, the cells were lysed in 250 µL nuclear lysis buffer (50 mM Tris.HCl, pH 8.1; 10 mM EDTA, pH 8.0; 1% SDS) to obtain the crosslinked chromatin. Chromatin was sonicated using a Bioruptor (Diagenode) at “high” power for 20 min (30 s on and 30 s off, for 20 cycles) at 4 °C. Chromatin from animals within the same treatment group was pooled (5 animals per pool) and precleared using protein A agarose beads (25%, diluted in ChIP dilution buffer; Protein A Agarose/Salmon Sperm DNA, Millipore, 16-157). Following preclearing, chromatin was distributed over separate tubes for incubation with antibodies. Chromatin corresponding to approximately 200.000 microglia per ChIP was incubated overnight at 4 °C (final buffer composition during antibody incubation was 0.1% SDS; 1% Triton-X-100; 2.4 mM EDTA; 20 mMTris.HCl, pH 8.1; 150 mM NaCl) with antibodies for specific histone modifications (the information of antibodies is available in Table 2, the specificity of antibodies have been checked for H3K27me3 peptides, the information of these peptides is listed in Table 2). The chromatin incubated with IgG was used as negative control while the chromatin saved without antibody incubation served as input. The next day, immune complexes were precipitated with 80 µL protein A beads (25%) for 2 h at 4 °C, washed by low salt wash buffer (150 mM NaCl; 0.1% SDS; 1% Triton-x-100; 2 mM EDTA, pH 8.0; 20 mM Tris.HCl, pH 8.1), high salt wash buffer (500 mM NaCl; 0.1% SDS; 1% Triton-x-100; 2 mM EDTA, pH 8.0; 20 mM Tris.HCl, pH 8.1), LiCl wash buffer (0.25 M LiCl; 1% NP-40; 1% Na-deoxycholate; 1 mM EDTA; 10 mM Tris.HCl, pH 8.1), and TE (10 mM Tris.HCl, pH 8.0; 1 mM EDTA, pH 8.0). After the chromatin was eluted from the beads, the precipitated chromatin was de-crosslinked overnight at 65 °C. Afterwards, RNase A (ThermoFisher, EN0531) and Proteinase K (Sigma, P2308) were added. Finally, the DNA was purified by GeneJET PCR purification kit (ThermoFisher, k0701).

**Table 2:** Antibodies used for ChIP

Antibody	Supplier	Full name	cat #	lot #
H3K4me1	Abcam	Anti-Histone H3 (mono-methyl K4) antibody—ChIP Grade	Ab8895	GR193737-1
H3K4me3	Millipore	Anti-trimethyl-Histone H3 (Lys4)	07-473	2117175
H3K27ac	Abcam	Anti-Histone H3 (acetyl K27) antibody—ChIP Grade	Ab4729	GR200563-1
H3K27me3	Millipore	ChIP Ab + ^tm^ Trimethyl-Histone H3 (Lys27)	17-622	2325081

Sequencing libraries were generated from the purified DNA by MicroPlex Library Preparation Kit v1 × 12 (Diagenode, C05010010) for tolerized samples or MicroPlex Library Preparation Kit v2 × 12 (Diagenode, C05010012) in case of primed samples. The libraries were quantified by Agilent 2100 Bioanalyzer, pooled and sequenced with a HiSeq Rapid SBS kit (50 cycles, Illumina, FC-402-4022) using single reads on a HiSeq 2500 (Illumina).

### ATAC-sequencing

ATAC-sequencing libraries were generated using Nextera^®^ DNA Sample Preparation Kit (Illumina, FC-121-1030) following the methods described by [66, 67]. A total number of 80,000 microglia were pooled from two animals (40,000 cells from each) and collected in Eppendorf tubes containing 300 µL medium A. Cells were pelleted by centrifugation (10 min, 4 °C, 500×*g*), resuspended in 50 μL of cold lysis buffer (10 mM Tris–HCl, pH 7.4, 10 mM NaCl, 3 mM MgCl_2_, 0.1% IGEPAL CA-630) and immediately centrifuged as before. Next, nuclei were resuspended in 50 μL transposition reaction mix (1 × TD reaction buffer, 2.5 μL TN5 transposase) and incubated at 37 °C for 30 min. Immediately following transposition, the DNA was purified using a minElute PCR purification kit (Qiagen, 28004) following the manufacturer’s instructions. The transposed DNA fragments were further amplified and barcoded [66, 67] and purified with a ChIP DNA Clean and Concentrator kit (Zymo, D5205). The fragments were run on 2% E-Gel™ EX agarose gels (Thermo Fisher scientific, G521802) and 150–600 bp fragments were excised, followed by purification with Zymoclean™ Gel DNA Recovery Kit (Zymo, D4007). Library concentration was determined with an Agilent 2100 Bioanalyzer after which 8 samples were pooled and sequenced using HiSeq Rapid SBS Kit v2 (50 cycles) using paired end reads on a HiSeq2500 (Illumina).

### ChIP- and ATAC-sequencing analysis

ATAC and ChIP samples were aligned to the *Mus musculus* genome (mm10/GRCm38) with the use of Bowtie 2 (v2.3.5.1 [68]) using the very-sensitive flag. Bamsort and bammarkduplicates from biobambam2 tools (v2.0.95) were used to sort the aligned files and to remove duplicated reads. Samtools (v.1.1.0, [69]) was used to remove low quality (*q* < 30) and blacklisted alignments. For Chip-seq data, peak calling was performed using the JetBrains SPAN peak analyzer (v.0.11.0) using default parameters, which were later manually refined upon visual inspection using the JetBrains JBR browser (v.1.0 beta) on each sample. BigWig files were generated using deepTools bamCoverage (v.3.5.0) with RPGC normalization. ATAC-seq peaks were called using Genrich (v.0.5) with the ATAC-seq mode (-j switch), and -p parameter set to 0.01. Differential peak calling for ChIP- and ATAC-seq were performed with manorm (v.1.3.0, [70]). The annotation of differential peaks was performed with the annotatePeaks function from R/Bioconductor ChIPseeker package (v1.24.0, [71]).

Analysis of differential transcription factor binding sites accessibility and classification of transcription factors into activators, repressors or undetermined was performed with the diffTF package (v1.7.1, [72]) based on ATAC- and RNA-seq data.

ChIP- and ATAC-seq peaks are visualized with the JetBrains SPAN peak analyzer.

The heatmap in Fig. 5c is based on the diffTF output in Supplemental file 6 & 8. The row z-score was calculated from weighted mean differences of ATAC peaks from the indicated comparison. Following, in each comparison non-significant differential peaks (adjusted *P* value > 0.001) and TF classified as ‘not-expressed’ were omitted. The row z-scores of significant differential accessible regions (adjusted *P* value > 0.001) of putative transcriptional activators, repressors and undetermined TFs are visualized in a heatmap assembled with the ‘Heatmap’ function of the ComplexHeatmap package (v2.4.3 [73]).

## Results

### LPS desensitization and accelerated aging result in distinct transcriptional responses in microglia

**Fig. 1:**
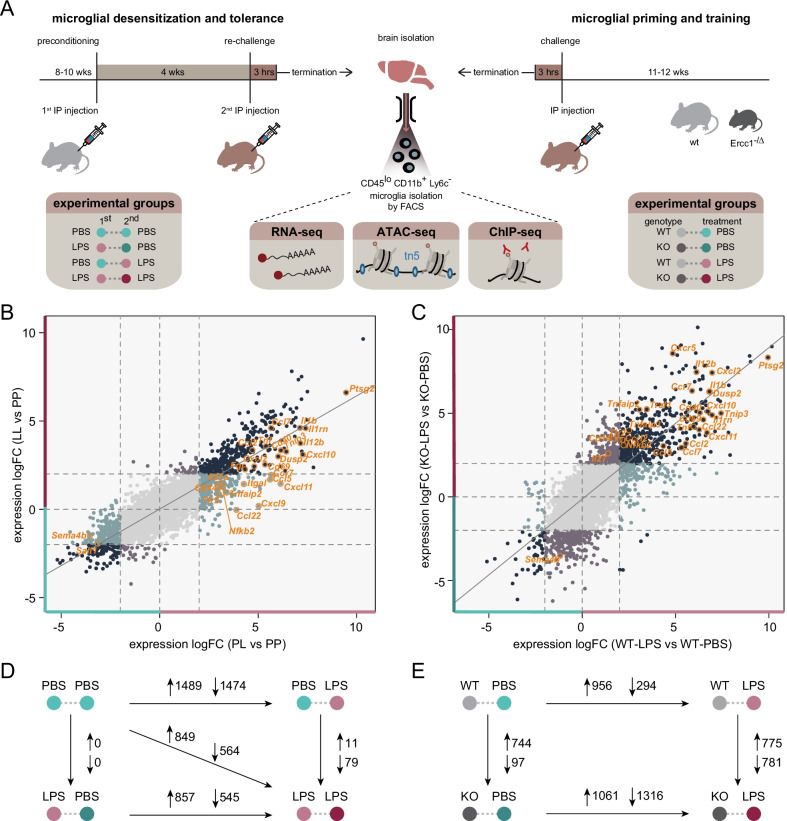
LPS desensitization and accelerated aging result in distinct changes of the microglia immune response. **a** Graphic representation of the mouse models and treatment groups. A pure microglia population was isolated by FACS and subjected to RNA, ATAC, and ChIP-sequencing analysis. **b**, **c** Four-way plots depicting changes in gene expression in microglia isolated from LPS-injected naive and pre-conditioned mice (*n* = 3 per experimental condition) (**b**) and *Ercc1*^*Δ/ko*^ and control mice (*n* = 3 per experimental condition) (**c**). Every gene is represented by an individual dot. Differentially expressed genes (LogFC > 2) are labeled with different colors indicating their respective expression changes. Dark blue dots indicate genes differentially expressed in both comparisons; turquoise (PL versus PP and WT-LPS versus WT-PBS) and lavender (LL versus PP and KO-LPS versus KO-PBS) dots represent genes differentially expressed in one of the comparisons. Several relevant genes are highlighted. **d**, **e** The number of differentially expressed genes (LogFC > 1 and FDR < 0.01) between treatment groups in the endotoxin tolerance (**d**) and *Ercc1*^*Δ/ko*^-induced microglia priming models (**e**). Upward arrows indicate increased gene expression, downward arrows indicate decreased gene expression in the condition where the large arrow points to

In mouse, previous data indicated two distinct microglia functional states of ‘desensitization’ induced by an intraperitoneal LPS challenge [17] and ‘priming’ during accelerated aging resulting from deficiency of the DNA-damage repair protein Ercc1 [14]. These different functional states can be unmasked by a (secondary) LPS stimulus resulting in a ‘tolerant’ or ‘trained’ immune response, respectively, and were so far characterized based on the analysis of limited sets of genes by qPCR [14, 17]. For several tested inflammatory genes, such as *Il1b*, *Tnf*, and *Il6*, the initial stimulus determined whether microglia show a dampened or enhanced response to (secondary) LPS treatment. However, the genome-wide transcriptional remodeling in desensitized and primed microglia and its effect on responsiveness to future inflammatory exposure are unknown. Therefore, we performed RNA-sequencing on acutely isolated, FACS-purified microglia (Additional file 1: Fig. S1) from mice that were either recurrently treated with LPS with a 1-month interval, or from *Ercc1*^*Δ/ko*^ mice that were stimulated with LPS near the end of their lifespan at 10–12 weeks of age (Fig. 1A).

For the tolerance model, we analyzed four treatment groups: the controls that were treated with PBS twice (PP), mice that were treated with LPS and after 1 month with PBS (LP) to investigate desensitization, mice treated with PBS followed by LPS after 1 month to determine the acute response to LPS (PL) and mice that were treated with LPS twice with a 1-month interval between challenges (LL) to identify the tolerant response (Fig. 1A). As has been previously described, after LPS injection, the animals experienced temporal peripheral inflammation, sickness behavior and weight-loss due to decreased food and water intake [74] (Additional file 2: Fig. S2A, S2B). I.p. injection of LPS resulted in significant changes in gene expression in microglia after 3 h (Fig. 1B, D; Additional file 2: Fig. S2C, Additional file 5). After 1 month, this initial response to LPS had subsided and in terms of the transcriptional program, no significant differences were observed between the PP and LP groups (Fig. 1D; Additional file 2: Fig. S2C, Additional file 5). However, when mice were challenged with LPS for a second time, the response was different from the initial response (Additional file 2: Fig. S2C) and many genes were significantly differentially expressed between PL and LL conditions (Fig. 1B, D; Additional file 5).

For the microglia priming model, both the *Ercc1*^*Δ/ko*^ mice and their control littermates were treated with PBS (WT-PBS, KO-PBS) to identify priming effects or with LPS (WT-LPS, KO-LPS) to identify training. As we observed previously, deletion of *Ercc1* in itself results in significant changes in gene expression (Fig. 1E; Additional file 2: Fig. S2D, Additional file 6). However, when *Ercc1*^*Δ/ko*^ mice were treated with LPS, the difference between microglia from control and knockout mice was much more pronounced (Fig. 1B, E; Additional file 2: Fig. S2D, Additional file 5).

The response to an acute LPS stimulus was highly similar in mice of the tolerance (C57BL/6J) and priming (FVB/C57BL/6J) model. Nevertheless, it cannot be fully excluded that the LPS response is slightly affected by the genetic backgrounds of the two mouse strains used in this study.

Many genes differentially expressed between PP versus LP and WT-PBS versus WT-LPS showed very similar changes in expression in response to LPS, after ranking them based on expression level and comparing rank positions between the two groups (Additional file 2: Fig. S2E). With our RNA-sequencing dataset, we confirmed several of our previous findings [14, 27], and replenish this information with complete gene expression profiles of the desensitized and primed microglia phenotype. The opposite regulation of the pro-inflammatory genes *Il1b* in tolerant (LL) and trained (KO-LPS) microglia (Additional file 2: Fig. S2F, S2G) was confirmed. In addition, primed microglia (KO-PBS) showed increased expression of genes belonging to the ‘primed’ gene hub [27], including *Clec7a* and *Axl* when compared to control animals (WT-PBS, Additional file 2: Fig. S2G).

### Genes with distinct transcriptional responses to LPS have different biological functions

**Fig. 2:**
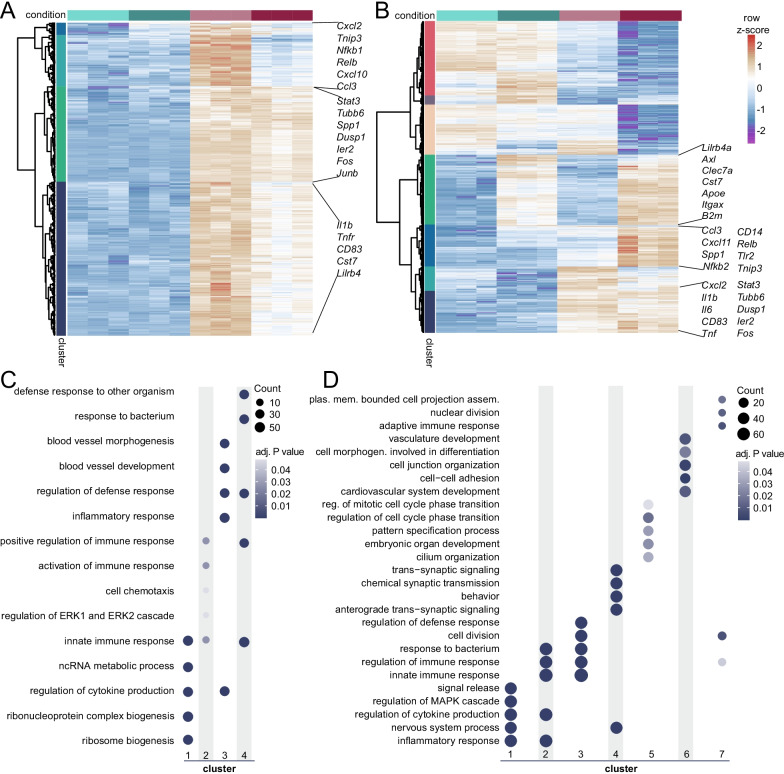
Identification of gene clusters with distinct transcriptional programs in desensitized and primed microglia. **a**, **b** Heatmaps with Manhattan distance-based hierarchical clustering analysis of upregulated genes in response to LPS in microglia of C57/BL6 mice three hours after i.p. injection with LPS (LogFC > 1 and FDR < 0.01, PL versus PP). Four main clusters are identified, containing tolerized genes (cluster 2 and 4) and responsive genes (cluster 1 and 3) that show distinct activity to LPS re-stimulation. **b** Heatmap with Manhattan distance-based hierarchical clustering analysis of all genes differentially expressed between *Ercc1*^*Δ/ko*^ (KO) and *Ercc1*^*wt/ko*^, *Ercc1*^*wt/Δ*^*, Ercc1*^*wt/wt*^ (WT) mice with or without LPS injection (n = 3 per experimental condition). Seven clusters are identified, including two clusters of genes primed and trained to LPS treatment in KO mice (cluster 3 and 2). **c**, **d** Top 5 GO annotations, based on gene count per GO term, of responsive (cluster 1, 3) and tolerized (cluster 2, 4) gene clusters (**c**) and the 7 clusters identified in *Ercc1*^*Δ/ko*^ microglia (**d**).

Following 3 h of LPS exposure (PL versus PP, LogFC > 1, FDR < 0.01), 1489 genes showed increased expression (Figs. 1D, 2A) while 1474 genes were downregulated in microglia (Fig. 1D; Additional file 3: S3A). Generally, LPS-induced genes were involved in various aspects of the immune response (Additional file 3: Fig. S3B, Additional file 7), while genes downregulated by LPS were involved in multiple biological processes (Additional file 3: Fig. S3C, Additional file 7). Of note, in the LPS-downregulated genes, the association with biological processes showed a lower level of significance than the GO terms associated with LPS-upregulated genes (Additional file 3: Fig. S3B, S3C; Additional file 7).

Focusing on LPS-induced genes, out of 1489 genes, 1187 responded similarly in case of re-stimulation with LPS (cluster 1 and 3), while 302 showed a reduced response to a second LPS challenge (cluster 2 and 4, Fig. 2A). Processes uniquely associated with the 1187 responsive genes were ‘ribosome biogenesis’, ‘regulation of cytokine production’, and ‘inflammatory response’, while ‘positive regulation of immune response’ and ‘response to bacterium’ were particularly associated with the 302 tolerized genes (Fig. 2C, Additional file 7). This is in line with the finding that tolerant monocytes/macrophages are impaired in their ability to produce pro-inflammatory cytokines [6, 75], but are capable of expressing genes involved in damaging or killing pathogens, so-called antimicrobial effectors. These data suggest that an i.p. injection with LPS initially induces a major immune response in microglia, which then results in the establishment of long-term innate immune tolerance that is characterized by a significantly reduced transcriptional response to secondary LPS treatment.

### Primed microglia have a genome-wide exaggerated response to LPS treatment

To gain insight into the biological processes affected by *Ercc1* deletion in microglia from unstimulated and LPS-treated mice, Manhattan distance-based hierarchical clustering analysis of genes followed by gene ontology analysis per cluster was performed (Fig. 2B, D; Additional file 8). Seven clusters were identified containing genes that were altered by Ercc1 deletion (KO). Genes of clusters 5 are similarly affected in WT and KO microglia and downregulated in both genotypes after LPS treatment. GO terms associated with these genes included ‘regulation of cell cycle phase transition’, and ‘pattern specification process’. The expression of genes in cluster 7 are induced in KO compared to WT microglia and are depleted in both conditions after LPS treatment. These genes are involved in processes like ‘nuclear division’, ‘cell division’ and ‘the immune response’. Cluster 6 contain genes that are upregulated in microglia of PBS- and LPS-treated WT compared to KO mice. These genes are associated with ‘cell junction organization’ and ‘cell–cell adhesion’.

Cluster 3 contains genes that were induced in PBS-treated and to a greater extent in LPS-treated KO compared to WT microglia. These primed genes are associated with GO terms ‘regulation of defense response’, ‘cell division’, ‘response to bacterium’ and ‘innate immune response’. Cluster 2 contains genes that were induced by LPS in KO and to a lesser extent in WT microglia and these genes were associated with GO terms such as ‘response to bacterium’, ‘innate immune response’ and ‘regulation of cytokine production’, underlining the trained immune response of KO microglia to LPS challenge. Cluster 4 contains genes that were induced by LPS in WT and to a lesser extent in KO microglia and these genes were associated with GO terms such as ‘trans-synaptic signaling, ‘chemical synaptic transmission’ and ‘nervous system process’. Finally, genes in cluster 1 are induced by LPS to a similar degree in WT and KO microglia and are associated with GO terms, like ‘signal release’, ‘regulation of cytokine production’ and ‘inflammatory response’ (Fig. 2D).

In agreement with our previous findings [14], also at a genome-wide level, Ercc1 deficiency generates an environment where microglia are more responsive to inflammatory stimuli, as evidenced by a large set of inflammatory genes whose expression is significantly increased in microglia upon LPS treatment of *Ercc1*^*Δ/ko*^ mice.

### Epigenetic remodeling in response to LPS desensitization and accelerated aging

The transcriptomes of microglia from PP and LP treated mice are almost identical, however, they respond very differently to re-stimulation with LPS (Fig. 2A; Additional file 2: Fig. S2C). Similarly, many genes that are not transcriptionally altered in Ercc1 deficient mice show an increased transcriptional response to LPS (Fig. 2B; Additional file 2: Fig. S2D). These data suggest that microglia have innate immune memory that is not secured in their transcriptome. Rather, similar to macrophages and as suggested by our previous analysis of the *Il1β* locus (Schaafsma et al. [17]), it is likely that epigenetic reprogramming is involved.

To gain insight in the genome-wide epigenetic changes induced by LPS desensitization and Ercc1 deficiency, we performed assay for transposase accessible chromatin-sequencing (ATAC-seq), which indiscriminately identifies open chromatin regions in the genome [66, 67], and chromatin immunoprecipitation-sequencing (ChIP-seq), which probes histones carrying specific posttranslational modifications [76, 77]. In case of the tolerance model, we used antibodies targeting H3K4me3 and H3K27Ac to identify transcription start sites (TSSs) and enhancers of actively transcribed genes, respectively. In *Ercc1*^*Δ/ko*^ mice, we also analyzed H3K4me3 and H3K27ac, and additionally H3K4me1 which together with H3K27ac marks active enhancers and the Polycomb-regulated H3K27me3 associated with transcriptional repression (Additional file 4: Fig. S4A).

Representative examples of chromatin accessibility and occupation, and RNA expression of individual tolerized (*Il1b*, *Tnf*, *Ccl3, Nfkb1* and *Relb,* Additional file 2: Fig. S2E, Additional file 4: Fig. S4B) and primed/trained (*Il1b, Ccl3, Cxcl11*, *Clec7a* and *Axl,* Additional file 2: Fig. S2F, Additional file 4: Fig. S4C) genes are depicted and indicate dynamic regulation of epigenetic signatures associated with changes in gene expression levels.

### Epigenetic characterization of tolerized genes

In order to determine which chromatin characteristics correspond to the transcriptional changes induced by LPS, we identified regions in the genome with significant differences in chromatin accessibility or histone modifications. Differential peaks were classified as promoters when they were located within 1000 bp downstream and 1000 bp upstream of a TSS of the nearest gene and as enhancers when being located distal of this region. To integrate RNA-, ATAC-, and ChIP-seq data, the differentially expressed genes (logFC) were correlated to differentially regulated chromatin regions (*M*-value) within one comparison.

**Fig. 3:**
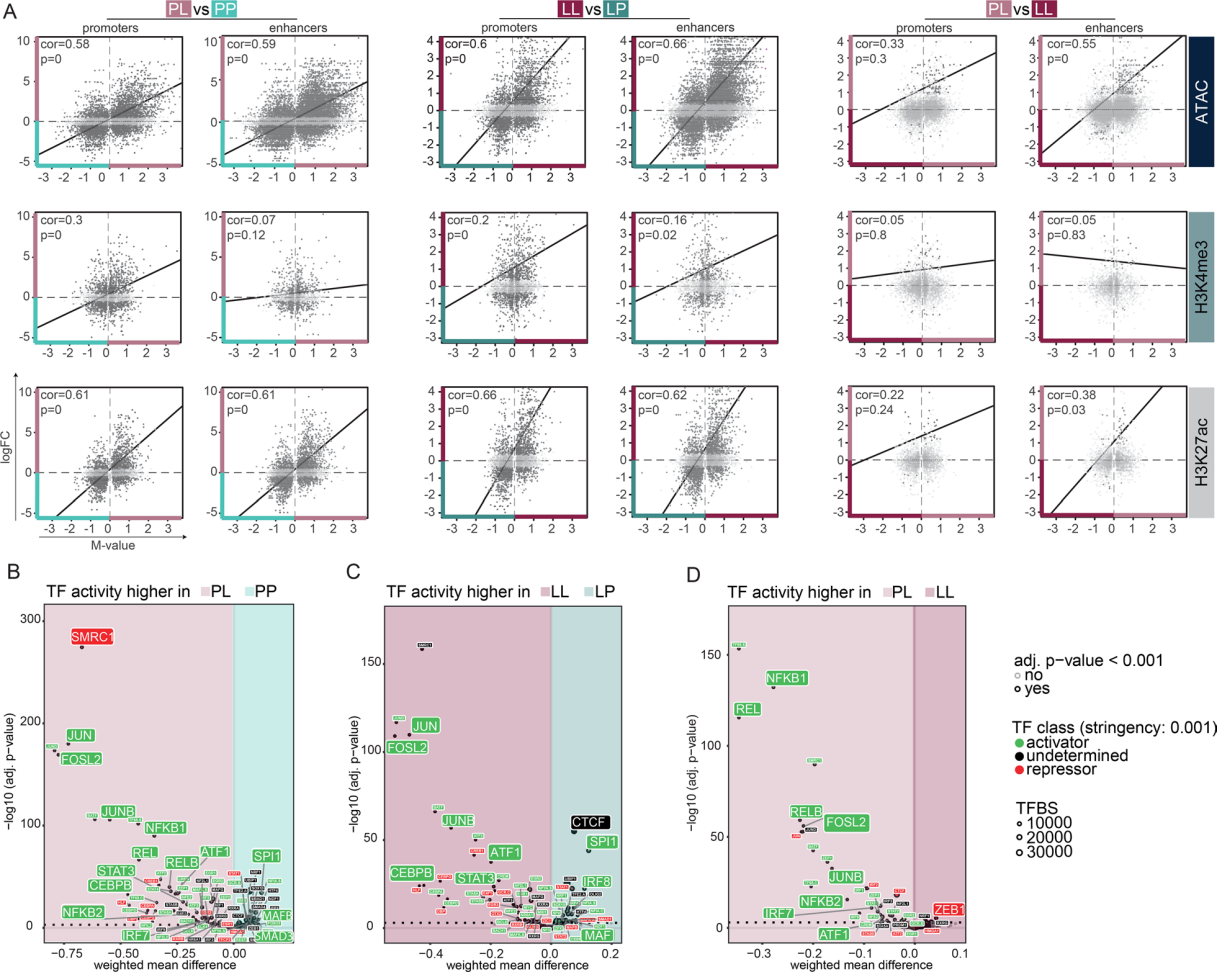
The LPS response in naive and desensitized microglia is defined by enhancer signatures of transcriptional permissive marks. **a** Scatterplots depicting the correlation of differentially expressed genes (logFC) with corresponding differential ATAC, H3K4me3 or H3K27ac peaks (M-value) at promoters (within 2 kb of the nearest TSS) or enhancers (distal to promoters) between PL versus PP (left panel), LL versus LP (middle panel), PL versus LL (right panel). Each dot represents a differentially expressed gene that is associated with a significant differential chromatin peak (FDR < 0.0)1 in the given comparison. Light gray-colored dots indicate non-significant gene expression differences (FDR > 0.01). **b–d** Transcription factor binding site analysis generated by diffTF to identify critical regulators for different gene sets based on ATAC- and RNA-seq data. Volcano plots depicting weighted mean difference of accessible TFBS between PL versus PP (**b**), LL versus LP (**c**), or PL versus LL (**d**). The color of each TF indicates its classification into an activator (green), a repressor (red) or undetermined (black) based on correlation of TFBS accessibility with RNA expression of the TF. *FC* fold change, *TF* transcription factor, *TFBS* transcription factor binding site

Similar to what has been described in macrophages [5, 78, 79], in microglia H3K4me3 already marks TLR4-responsive promoters prior to LPS stimulation (Additional file 4: Fig. S4B). Irrespective whether microglia are exposed to LPS for the first or the second time, genes which are expressed in response to LPS are, except for a small group of tolerized genes, largely overlapping (Fig. 2A). LPS-induced gene expression significantly correlates with ATAC, H3K4me3, H3K27Ac peak enrichment, associated with permissive gene expression, in promotors and enhancers of microglia from PL compared to PP (Fig. 3A left panel, Additional file 9) and LL compared to LP-treated mice (Fig. 3A middle panel, Additional file 9). In line with the fact that H3K4me3 is generally associated with promoters, LPS-induced gene expression seems to only significantly correlate with enrichment of this mark in promoters but not enhancers in the PL versus PP comparison (Fig. 3A left panel, Additional file 9).

Tolerized genes are characterized by increased expression after the primary LPS challenge (PL) and reduced induction after the secondary LPS challenge (LL) (Fig. 2A). When comparing the microglia response to primary and secondary LPS challenge (PL versus LL), the expression of the tolerant genes after primary LPS challenge significantly correlated with enrichment of ATAC and H3K27ac peaks at enhancers, but not promoters (Fig. 3A right panel, Additional file 9). This means vice versa that after secondary LPS challenge, tolerized genes were depleted in these activating expression-associated enhancer marks. The expression of tolerized genes was not significantly correlated to the promoter-associated histone mark H3K4me3. Together, these results indicate that the tolerized response of microglia to LPS seems to be mainly enhancer driven and, at least partially, explained by a loss of histone marks associated with active expression after secondary LPS challenge (Fig. 3 right panel, Additional file 9).

Transcription factors (TFs) are critical determinants of changes in both transcriptional and epigenetic programs that can be activated by signaling pathways. TFs are often part of large, multimeric protein complexes that also contain chromatin-modifying enzymes, and recruitment of TFs can result in local remodeling of the chromatin [80]. DiffTF was used to identify the TFs that might be involved in the differential chromatin regulation in tolerant microglia. Differential chromatin accessibility peaks (weighted mean difference) of putative TF binding sites (TFBS) between two conditions were identified. Next, this ATAC-seq data were integrated with RNA-seq data by correlating differential accessible peaks of putative TFBS to differential gene expression levels of a particular TF. This procedure is then repeated for each TF. Based on whether the correlation of TF activity and expression is positive or negative, TFs were classified as an activator or a repressor. Alternatively, when there was no correlation detected, the TF was classified as undetermined or the TF was not expressed (Berest et al. [72], Fig. 3B–D).

Genome-wide accessible chromatin regions, significantly enriched in naïve microglia (PP, Fig. 3B; Additional file 10), contain binding sites for the key myeloid TFs PU.1 (SPI1), IRF8 and MAFB, described to be crucial for adult mouse microglia transcriptional identity [22, 81]. In addition, naïve microglia are enriched in TFBS for SMAD3, an effector molecule downstream of TGFβ [82], which is critical for the microglia homeostatic signature [29]. Binding sites of homeostasis-associated TFs were lost and TFBS of known mediators of LPS-induced inflammatory pathways in macrophages/microglia [27, 78, 83] including the NF-κB TF family (NFKB1/2, REL/RELB, [84]), TFs involved in the immediate early response (IER; JUN, JUNB, FOSL2) and the interferon pathway (IRF TF family), STAT3, CEBPB [85–87], and the general activating transcription factor ATF1 were all detected to be enriched in microglia acutely challenged with LPS (PL versus PP, Fig. 3B; Additional file 10).

After a secondary LPS challenge, in tolerant microglia (LL vs. LP), many of these inflammatory-associated TFBS are still enriched, except those belonging to the NF-κB TF family, indicating that recruitment of these TFs specifically occurs after primary LPS challenge. This is also confirmed in the direct comparison of acutely stimulated versus tolerant microglia (PL vs. LL, Fig. 3D; Additional file 10). Furthermore, the enrichment of TFBS for SPI1, IRF8, CTCF and MAF, important for the homeostatic microglia transcriptome [22, 31], in desensitized microglia (LP vs. LL, Fig. 3C; Additional file 10) explains their naive-like transcriptome (Fig. 1D).

Many of the inflammatory-associated putative TFBS are depleted and the TFBS for the transcriptional repressor ZEB1, associated with suppression of immune active genes [88, 89], are enriched in tolerant microglia (LL) when compared to microglia of acutely LPS-challenged mice (PL, Fig. 3D; Additional file 10), possibly explaining the dampened expression of tolerized genes in LL microglia.

These data indicate that deposition of permissive chromatin marks drive the acute LPS-response of microglia, and loss of those, in particular surrounding TFBS of the NF-κB family, might at least partially explain the tolerized response of microglia to a secondary LPS-challenge.

### Epigenetic characterization of the priming response

**Fig. 4:**
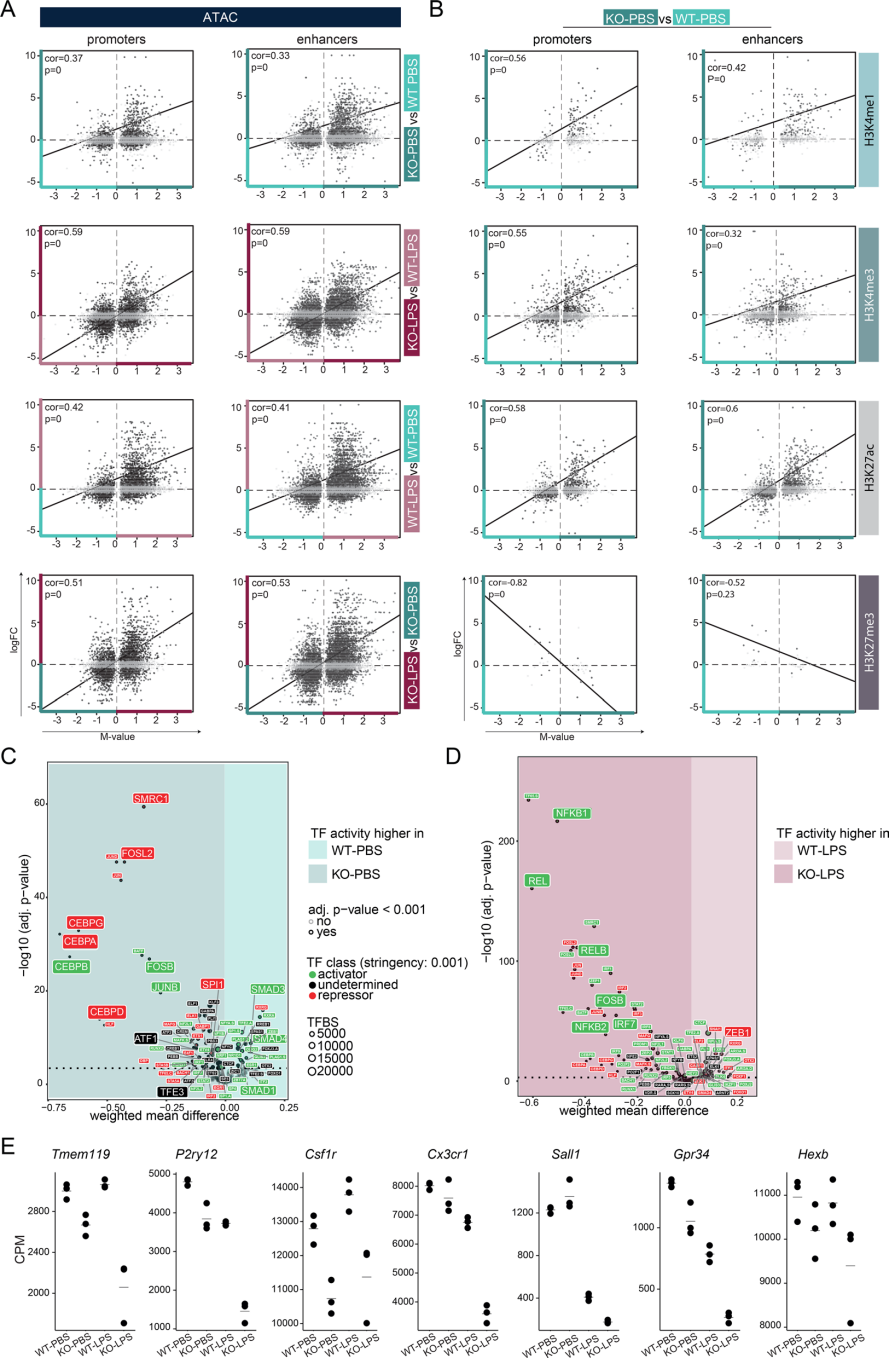
Enhancer and promoter signatures of transcriptional permissive marks regulate training in primed microglia. **a**, **b** Scatterplots depicting the correlation of differentially expressed genes (logFC) with corresponding differential ATAC peaks (M-value) in KO versus WT, LPS-treated KO versus LPS-treated WT, LPS-treated WT versus WT and LPS-treated KO versus KO microglia (**a**), and differential H3K4me1, H3K4me3, H3K27ac or H3K27me3 peaks (M-value) in KO versus WT microglia (**b**). The chromatin peaks are divided into promoters (within 2 kb of the nearest TSS) and enhancers (distal to promoters). Each dot represents a differentially expressed gene that is associated with a significantly differential chromatin peak (FDR < 0.01) in the given comparison. Gray color of dots indicates non-significant gene expression differences (logFC > 1, FDR > 0.01). **c**, **d** Transcription factor binding site analysis generated by diffTF to identify critical regulators for different gene sets based on ATAC- and RNA-seq data. Volcano plots depicting weighted mean difference of accessible TFBS between KO-PBS versus WT-PBS (**c**) and KO-LPS versus WT-LPS (**d**) microglia. The color of each TF indicates its classification into activator (green), repressor (red) or undetermined (black) based on correlation of TFBS accessibility with RNA expression of the TF. **e** Gene expression values (CPM, Additional file 6) of selected homeostatic microglia genes in the primed mouse model. Every dot depicts an individual animal (*n* = 3 per experimental condition). *CPM* counts per million reads, *TF* transcription factor, *TFBS* transcription factor binding site

In case of microglia priming, we also observed a general concordance between the transcriptional changes following Ercc1 deficiency and LPS challenge and the presence of permissive chromatin characteristics. Induction of gene expression by *Ercc1* KO or by LPS in both WT and KO microglia significantly correlated with increased chromatin accessibility in promoters as well as enhancers (Fig. 4A; Additional files 6, 11). In addition, compared to WT-PBS, many KO-induced genes are marked with significant enrichment of the permissive marks H3K27Ac and H3K4me3 (Fig. 4B). The expression of some of the KO-induced genes additionally correlates with H3K4me1 enrichment, which together with H3K27ac deposition is associated with active transcription [90]. Inversely, some of the genes whose expression is induced by Ercc1 deficiency are depleted in H3K27me3, which is associated with Polycomb-associated gene repression, at promoters of microglia from KO versus WT mice (Fig. 4B). Together, this indicates that the expression of primed genes in Ercc1 deficient mice might be driven by enriched chromatin characteristics associated with permissive transcription and depletion of repressive chromatin marks.

We next determined accessible conserved TFBS and corresponding expression of the TFs in microglia of (LPS-treated) Ercc1 deficient and WT mice. Compared to controls, SMAD1/3/4 binding sites are lost in microglia of KO mice (Fig. 4C; Additional file 12), which are involved in maintenance of the microglia homeostatic gene signature [29, 82]. Generally, immune activation of microglia results in the loss of the homeostatic signature [27, 40, 41, 91], and our data show that this is also true in primed microglia (Fig. 4E). Microglial TF motifs with increased chromatin accessibility upon Ercc1 deletion include TFs whose associated functions were previously attributed to primed microglia [14, 27], namely lysosomal biogenesis (TFE3, [92]), inflammation (CEBP TF family [85–87], IER TF family, ATF1) and proliferation (CEBP TF family, [93, 94]) (Fig. 4C; Additional file 12).

In contrast to microglia of LPS-treated WT mice, trained microglia of LPS-treated KO mice are enriched in accessible TF motifs for regulators with known roles in acute LPS-induced inflammation [27, 78, 83], including NFKB2 and REL/RELB, several members of the IRF TF family (IRF7, 8, 9), and IER-related TFs. In addition, ZEB1, associated with immune response suppression [88, 89], is depleted in trained microglia (Fig. 4D; Additional file 12). Together with the fact that homeostatic genes in microglia of LPS-treated KO mice are even further downregulated than in KO microglia (Fig. 4E), these results underline the training of microglia from KO mice.

These data suggest that Ercc1 depletion shapes a chromatin landscape that enables both the loss of the microglia homeostatic signature, and the gain of a transcriptional profile associated with inflammation, which is enhanced with LPS challenge.

### A large proportion of tolerized genes show an increased transcriptional response in primed and trained microglia

Both in tolerized (cluster 2 and 4, Fig. 2A) and primed/trained gene sets (cluster 1, 2 and 4, Fig. 2B), immune system processes were significantly enriched (Fig. 2C, D). We intersected these gene sets and not only were similar biological processes affected, but many of the differentially regulated genes were also shared.

**Fig. 5:**
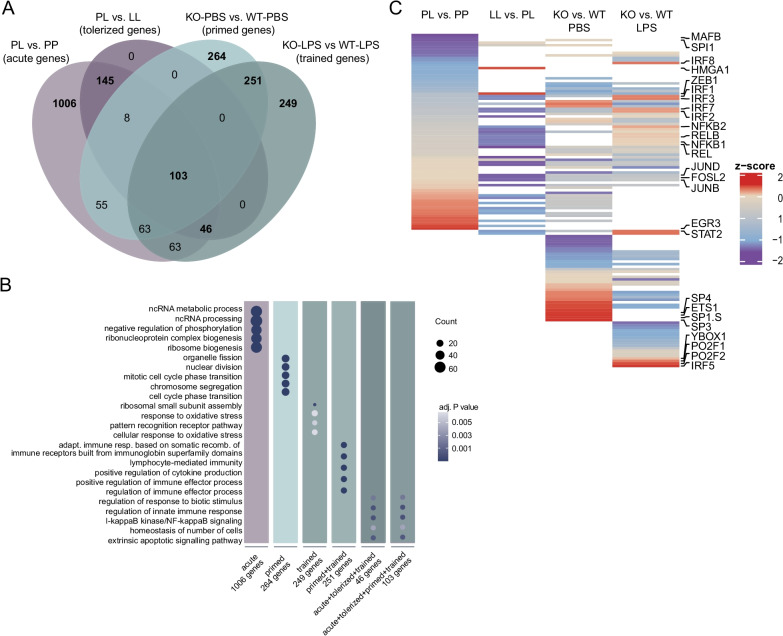
Inflammatory genes show distinct epigenetic regulation in ‘acute’, ‘tolerant’, ‘primed’ and ‘trained’ microglia. **a** Venn diagram of the enriched genes in acute (PL versus PP, light purple, Additional file 5), tolerized (clusters 2 and 4, dark purple, Additional file 7), primed (KO-PBS versus WT-PBS, mint, Additional file 6) and trained (KO-LPS versus WT-LPS, dark mint, Additional file 6) microglial response. **b** Dotplot depicting the GO terms associated with unique and overlapping gene sets of acute, tolerant, primed and trained microglia. The size of the dot represents the gene count per GO term and the color indicates the adjusted *P*-value. **c** Heat map depicting row z-scores of weighted mean differences (adjusted *P* value < 0.001) of ATAC peaks at loci of specific TF motifs in the indicated comparisons identified with diffTF (based on Figs. 3B, D, 4C, D; Additional files 10, 12). Only accessible TF motifs of activating, repressing and undetermined TF are displayed (not-expressed TFs were filtered out). White space indicates non-significant weighted mean differences of ATAC peaks at the given locus and/or not-expressed TFs in the indicated comparison.

Out of the 302 tolerized genes, 145 genes overlap with acute LPS response-induced genes and 46 showed a significantly higher expression level in microglia of LPS-treated mice and *Ercc1*^*Δ/ko*^ mice after LPS treatment. 264 and 249 genes were uniquely enriched in primed (KO-PBS versus WT-PBS) and trained (KO-LPS versus WT-LPS) microglia, respectively, and 251 genes overlapped between these conditions. Finally, 103 overlapping genes were enriched in acutely challenged, tolerized, primed as well as trained microglia (Fig. 5A; Additional file 13). Significantly associated biological processes within these gene sets were identified (Fig. 5B; Additional file 14). Genes involved in ‘organelle fission’, ‘nuclear division’ and ‘chromosome segregation’ were associated with and limited to primed microglia from Ercc1 deficient mice. The genes exclusive for training are involved in ‘response to oxidative stress’ and ‘ribosomal small subunit assembly’. The 251 genes that are shared between primed and trained microglia are associated with ‘positive regulation of cytokine production’ and ‘regulation of immune effector process’. The acute, tolerized and trained gene sets, with or without the primed gene set, share GO terms such as ‘regulation of innate immune response’, ‘NF-kappaB signaling’ and ‘regulation of apoptotic signaling pathway’.

In order to determine possible regulators of the opposing LPS response between tolerized and trained genes, motifs for TFBS in genomic regions with enriched chromatin accessibility were identified with diffTF in acute (PL versus PP), tolerant (LL versus PL), primed (KO-PBS versus WT-PBS) and trained (KO-LPS versus WT-LPS) microglia (Fig. 5C; Additional file 10, 12). The four identified microglial phenotypes (acute, tolerized, primed, trained) seem to be regulated by specific TF networks, explaining why gene sets, although being partially shared between some or all of the four phenotypes, are regulated in opposite directions.

Summarizing, the presented data indicate that microglia in vivo possess innate immune memory and that different types of stimuli, in this case Ercc1 deficiency or LPS, can leave epigenetic imprints which seem to influence the response towards a secondary challenge leading to microglia training or tolerance to LPS. Condition-specific epigenetic profiles seem to involve the activity of specific TF networks, which might drive the opposite regulation of shared genes in trained and tolerant microglia.

## Discussion

Monocytes and tissue-resident macrophages play important roles in development, metabolism and immunity, thereby contributing to the maintenance of homeostasis. Though they are innate immune cells, macrophages can retain information of past inflammatory events, resulting in an altered response to reinfection. Depending on the primary trigger, macrophages can become ‘tolerant’, showing hypo-responsiveness, or ‘trained’ with increased responsiveness to subsequent stimuli. Biologically, these mechanisms are generally thought to provide a survival advantage in case of trained immunity [95], while the refractory state of tolerant macrophages causes increased mortality [4]. However, these effects seem to be context-dependent and it was hypothesized that trained immunity might have deleterious consequences in autoimmune diseases (Arts, Joosten, et al. [96]), whereas tolerance can provide a protective mechanism limiting the toxic effects of prolonged inflammation [97].

Monocytes/macrophages undergo functional programming after exposure to microbial components [6, 8] and the associated genome-wide epigenetic characteristics of innate immune memory have been described over the past years [5, 6, 98–100]. These observations are thought to provide clues as to which pathways to target to reverse ‘tolerance’ or stimulate ‘training’ in a clinical setting.

The CNS parenchyma contains microglia, tissue-resident macrophages that fulfill highly specialized functions extending far beyond their innate immunological functions [9, 101]. Besides their different job-description that is attuned to their CNS environment, in contrast to some other tissue-derived macrophages, microglia also have a relatively long lifespan [48, 49, 101, 102]. Microglia longevity together with the long-lasting nature of epigenetic mechanisms can have drastic effects on brain functioning and cognition.

In microglia, altered functional outcomes reminiscent of ‘tolerance’ and ‘training’ have been described and these mechanisms might contribute to poor cognitive outcomes in sepsis patients [103], the general aged population and neurodegeneration [2, 11, 36, 44, 104, 105]. Particularly, disease features in mouse AD and stroke models appear to be altered in animals where microglia were exposed to systemic inflammatory stimuli [11].

Many factors influence the extent to which a peripheral LPS injection induces a response in CNS resident macrophages, including microglia. These include the dose of LPS [106] the time between the LPS administration and analysis [107], the measured output parameter (morphological changes take more time to take shape compared to changes in RNA expression) and ambient temperature [108]. Here, we show that under our experimental conditions, exposure of microglia to an inflammatory challenge (LPS) or an environment of accelerated aging in vivo results in substantial transcriptional and epigenetic changes that impact on their future ability to mount an inflammatory response. In particular, we found that approximately 103 genes are oppositely regulated when ‘desensitized’ or ‘primed’ microglia are exposed to i.p. injection of LPS and that these genes are involved in inflammatory and apoptotic processes.

In the control situation, promoter and cis-regulatory elements associated with these inflammatory genes are characterized by a certain degree of chromatin accessibility, as well as H3K4me3 and H3K27Ac enrichment. In agreement with increased transcription of inflammatory genes in microglia from mice treated with LPS, these chromatin parameters were increased during the acute response. In case of tolerance, abundance of these marks is decreased after LPS re-exposure, which, at least partially, explains compromised induction of gene expression after secondary LPS challenge. Possibly, there is a second layer of gene expression repression by inhibitory histone marks. Previous data suggest a role for the inhibitory histone marks H3K9me2/3 in this context [17, 99]. The TF RELB has a recruiting role for H3K9me2/3 at the *Il1β* locus after LPS challenge, which leads to transcriptional repression of *Il1β* in response to a secondary LPS challenge [17, 45]. We identified enriched accessible binding motifs for REL and RELB in PL versus LL microglia genome-wide, indicating that a primary LPS challenge might lead to recruitment of REL/RELB at regulatory elements of tolerized genes and might inhibit gene expression upon secondary LPS challenge through recruitment of H3K9me2/3. However, this hypothesis needs to be confirmed in future ChIP-sequencing experiments.

In case of priming, gene sets involved in the immune response and cell division were enriched in *Ercc1*^*Δ/ko*^ microglia as well as after an LPS exposure. This data is substantiated by earlier findings showing an increase in the number of Ki67-positive Iba1 microglia in *Ercc1*^*Δ/ko*^ mice and increased phagocytotic activity and production of reactive oxygen species of LPS-challenged *Ercc1*^*Δ/ko*^ microglia [14]. Next to gene expression changes, the continuous exposure to an aging environment results in increased chromatin accessibility as well as H3K4me3 and H3K27Ac enrichment. SMAD binding elements are known to act collaboratively with PU.1 and other TFs to facilitate transcription of the homeostatic microglia signature [109]. In the accelerated aging model, chromatin signatures associated with active gene expression are less associated with SMAD binding elements. This is accompanied by a decrease in expression of homeostatic microglia signature genes in *Ercc1*^*Δ/ko*^ microglia, especially following LPS treatment. Microglia priming in this model is caused by neuronal genotoxic stress, since only Ercc1 deficiency in neurons, but not astrocytes and microglia induced microglia priming [14, 110]. While active marks on promoters and enhancers correlate with increased expression, the Polycomb regulated repressive mark H3K27me3 is lost in some associated genes whose expression is increased in *Ercc1*^*Δ/ko*^ microglia. Loss of the Polycomb mark H3K27me3 could be a critical determinant of cellular identity and function of primed microglia as the Polycomb repressive complex 2 (PRC2) is involved in maintenance of homeostatic microglia identity in different CNS brain regions. Loss of PRC2 activity in microglia resulted in aberrant gene expression and altered functionality [111].

Microglia training was previously observed in an AD amyloid mouse model, where an LPS challenge administered prior to the onset of AD pathology caused exacerbation of β-amyloidosis [11]. Although the hyper-responsive nature of microglia to two stimuli seems to be comparable in these two studies, the underlying molecular mechanisms might be different due to the fact that the LPS stimulus and AD pathology were separated by a non-inflammatory phase [11], while persistent microglial activation is present in *Ercc1*^*Δ/ko*^ mice.

Though the genes involved in tolerance and training are overlapping, the fact that the chromatin composition in these regions is diverse, suggests the involvement of distinct protein complexes and epigenetic enzymes. Summarizing, different molecular pathways and different epigenetic mechanisms regulate the behavior of inflammatory genes in ‘tolerant’ or ‘trained’ microglia.

## Conclusion

Our data provide evidence that at least one type of macrophage, the CNS endogenous microglia, in vivo can adopt transcriptional and epigenetic programs that contribute to the establishment of different functional phenotypes and thereby influence neuroinflammation in the long term.

## Supplementary Information

**Additional file 1: Fig. S1** related to Fig. 1, FACS sorting of microglia. **a,** Single, viable microglia are isolated using side scatter and forward scatter parameters, followed by exclusion of DAPI^pos^ (dead) events. Further purification was done by exclusion of Ly-6C^pos^ CNS macrophages. **b**, CD11b^pos^ and CD45^int^ microglia were sorted.

**Additional file 2: Fig. S2 **related to Fig. 1, RNA-sequencing of desensitized/tolerant and primed/trained microglia. **a, b,** Average (**a**) and individual (**b**) bodyweight (gram) before (Day -3), on the day of and just before the LPS injection (Day 0) and up to 25 days after LPS injection. **c, d,** PCA-plots of RNA-seq data of microglia in the LPS desensitization tolerance (**c**) and Ercc1-induced priming (**d**) mouse models. Every dot depicts an individual animal (n = 3 per experimental condition). **e,** Volcano plots illustrating the similarity in the acute LPS response in microglia from naive mice. Dots represent log fold change (LogFC) of differential expressed genes between PL and PP (**e**). Genes were ranked according to their expression level and based on that classified into percentiles. Next, for each gene of the two comparisons, the delta percentile was calculated and indicated as colors in the volcano plot, where light blue indicates similar and dark blue indicates deviant expression between the indicated conditions. Gray dots indicate gene expression differences with logFC < 1 and adjusted P values > 0.01. **f, g,** Gene expression values (CPM) of genes in the tolerance (**f**) or priming (**g**) mouse model. Every dot depicts an individual animal (n = 3 per experimental condition). CPM = counts per million reads, g = gram

**Additional file 3: Fig. S3. **Related to Fig. 2, LPS-downregulated genes and associated GO terms. **a,** Heatmap with Manhattan distance-based hierarchical clustering analysis of downregulated genes in response to LPS in microglia of C57BL/6 mice (n = 3 per experimental condition) three hours after i.p. injection with LPS (LogFC > 1 and FDR < 0.01, PP versus PL). **b, c,** Gene ontology (GO) analysis of genes upregulated (**b**) and downregulated (**c**) 3 h after LPS challenge in microglia of C57BL/6 mice. Based on gene count per GO term, the top 20 GO terms were identified. The size of the dot represents the gene count per GO term and the color indicates the adjusted P value.

**Additional file 4: Fig. S4. **Related to Figs. 3 and 4, ATAC- and ChIP-sequencing peak enrichment at representative desensitized and primed gene loci. **a,** Experimental strategy for the analysis of chromatin accessibility, and occupation by histone modifications. H3K4me3 and H3K27ac were analyzed in ‘desensitized’ and ‘tolerant’ microglia. H3K4me1, H3K4me3 and H3K27ac were determined in ‘primed’ microglia. **b, c**, Tracks of ATAC and indicated histone marks sequencing data of representative desensitized/tolerant (**b**) and primed/trained (**c**) genes. For ChIP, chromatin of 5 mice per experimental group was pooled; for ATAC, microglia (80,000 total) from 2 mice per experimental group were pooled. Tracks were visualized using JetBrains SPAN peak analyzer. Gene expression of these genes are shown in S2F and S2G.

**Additional file 5: **Count table and differentially expressed genes for all comparisons in the tolerance mouse model (related to Figs. 1 and 3).

**Additional file 6: **Count table and differentially expressed genes for all comparisons in the primed mouse model (related to Figs. 1 and 4).

**Additional file 7: **Genes and associated gene ontology terms for each cluster identified in the tolerance model (related to Figs. 2 and S3).

**Additional file 8: **Genes and associated gene ontology terms for each cluster identified in the priming mouse model (related to Figs. 2 and S3).

**Additional file 9: **Annotated differential ATAC, H3K4me3 and H3K27ac peaks in microglia of PL versus PP, PL versus LL and LL versus LP mice (related to Fig. 3A, 3B, 3C).

**Additional file 10: **TFBS of differential ATAC peaks in microglia of PP versus PL, LL versus PL and LP versus LL mice and classification of TFs based on correlation of TFBS peaks with TF target gene expression (related to Figs. 3D, 3E, 3F, 5C).

**Additional file 11: **Annotated differential ATAC, H3K4me1, H3K4me3, H3K27ac and H3K27ac peaks in microglia of KO-PBS versus WT-PBS, KO-LPS versus WT-LPS, WT-LPS versus WT-PBS and KO-LPS versus WT-PBS mice (related to Fig. 4A, 4B).

**Additional file 12: **TFBS of differential ATAC peaks in microglia of WT-PBS versus KO-PBS and WT-LPS versus KO-LPS mice and classification of TFs based on correlation of TFBS peaks with TF target gene expression (related to Figs. 4D, 4E, 5C).

**Additional file 13: **Lists of genes uniquely or overlappingly enriched in acute, tolerized, primed and trained microglia (related to Fig. 5A).

**Additional file 14: **GO terms associated with genes uniquely or overlappingly enriched in acute, tolerized, primed and trained microglia (related to Fig. 5B).

## Data Availability

All next-generation sequencing data can be viewed at NCBI GEO under accession number GSE175578.

## Abbreviations

AD: Alzheimers' disease
ATAC: Assay for transposase-accessible chromatin
ChIP: Chromatin immunoprecipitation
CNS: Central nervous system
CPM: Counts per million
FACS: Fluorescence activated cell sorting
GO: Gene Ontology
IIM: Innate immune memory
IP: Intraperitoneal
KO: Knock out
logFC: Log fold change
LPS: Lipopolysaccharide
LL: LPS-LPS treatment
LP: LPS-PBS treatment
PBS: Phosphate buffered saline
PL: PBS-LPS treatment
PP: PBS-PBS treatment
qPCR: Quantitative polymerase chain reaction
RNA: Ribonucleic acid
TSS: Transcription start site
TF: Transcription factor
TFBS: Transcription factor binding site
WT: Wild type
