# Supplementary figures and images for: Epigenetic regulation of innate immune memory in microglia
Source: J Neuroinflammation. 2022 May 14;19:111. doi: 10.1186/s12974-022-02463-5 (PMC9107649; doi:10.1186/s12974-022-02463-5)

A

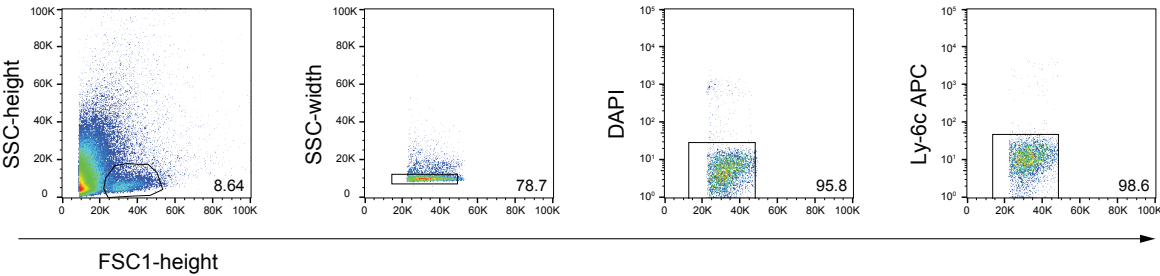

B

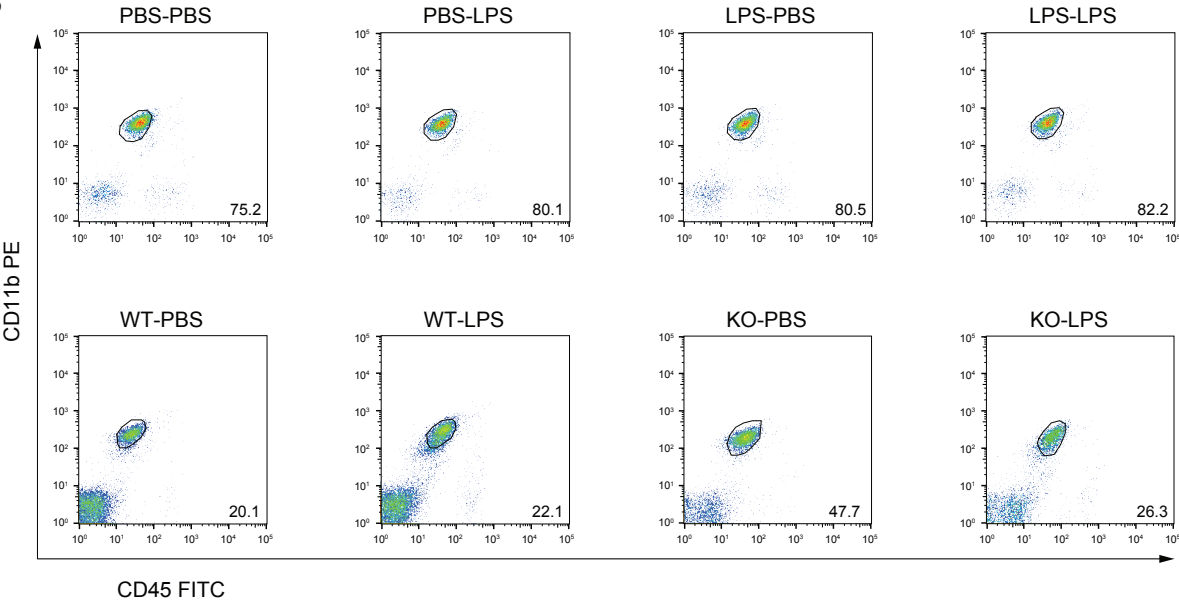

Supplement: Supplementary file 1 — Additional file 1: Fig. S1 related to Fig. 1, FACS sorting of microglia. a, Single, viable microglia are isolated using side scatter and forward scatter parameters, followed by exclusion of DAPIpos (dead) events. Further purification was done by exclusion of Ly-6Cpos CNS macrophages. b, CD11bpos and CD45int microglia were sorted. [file 12974_2022_2463_MOESM1_ESM.pdf]

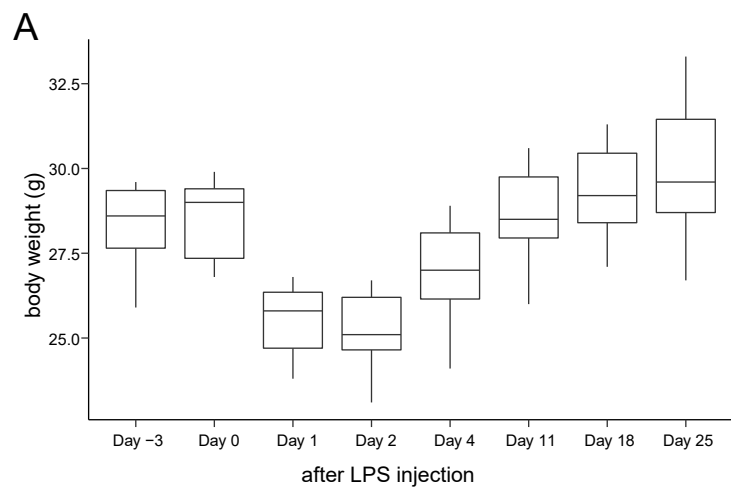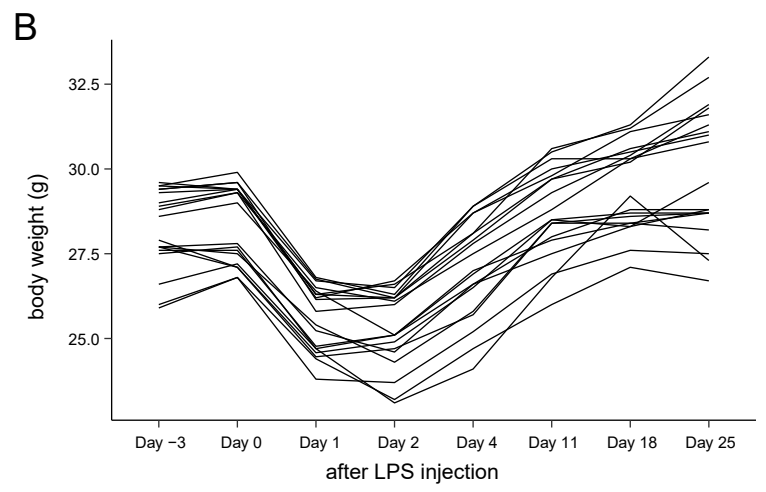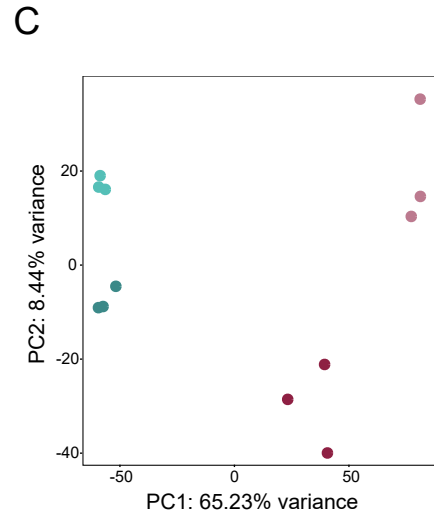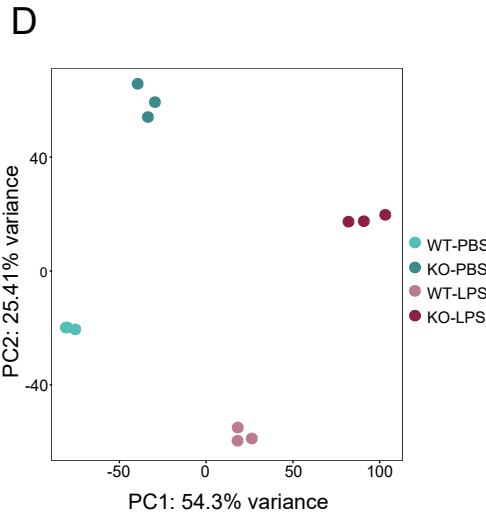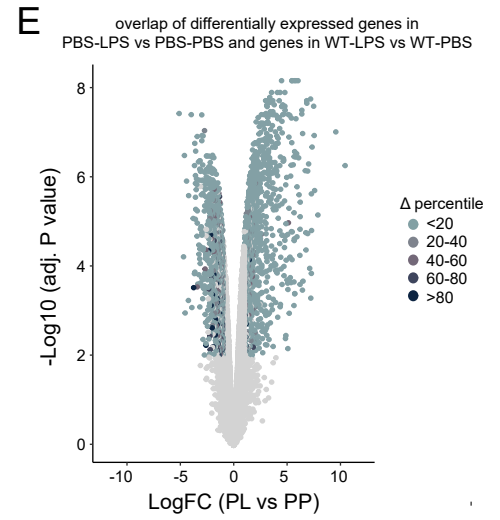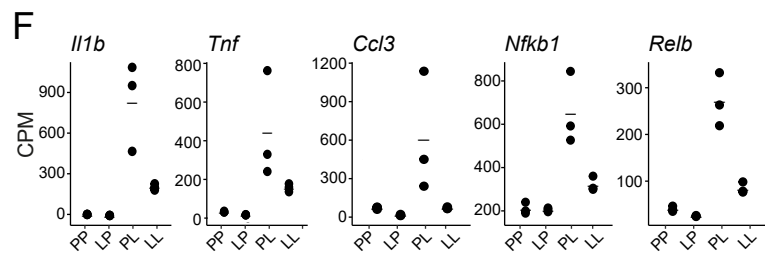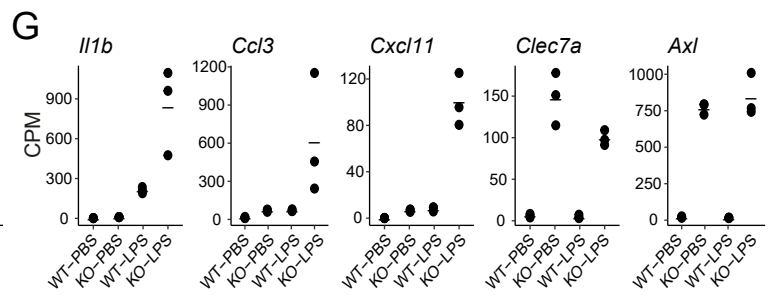

Supplement: Supplementary file 2 — Additional file 2: Fig. S2 related to Fig. 1, RNA-sequencing of desensitized/tolerant and primed/trained microglia. a, b, Average (a) and individual (b) bodyweight (gram) before (Day -3), on the day of and just before the LPS injection (Day 0) and up to 25 days after LPS injection. c, d, PCA-plots of RNA-seq data of microglia in the LPS desensitization tolerance (c) and Ercc1-induced priming (d) mouse models. Every dot depicts an individual animal (n = 3 per experimental condition). e, Volcano plots illustrating the similarity in the acute LPS response in microglia from naive mice. Dots represent log fold change (LogFC) of differential expressed genes between PL and PP (e). Genes were ranked according to their expression level and based on that classified into percentiles. Next, for each gene of the two comparisons, the delta percentile was calculated and indicated as colors in the volcano plot, where light blue indicates similar and dark blue indicates deviant expression between the indicated conditions. Gray dots indicate gene expression differences with logFC < 1 and adjusted P values > 0.01. f, g, Gene expression values (CPM) of genes in the tolerance (f) or priming (g) mouse model. Every dot depicts an individual animal (n = 3 per experimental condition). CPM = counts per million reads, g = gram [file 12974_2022_2463_MOESM2_ESM.pdf]

A

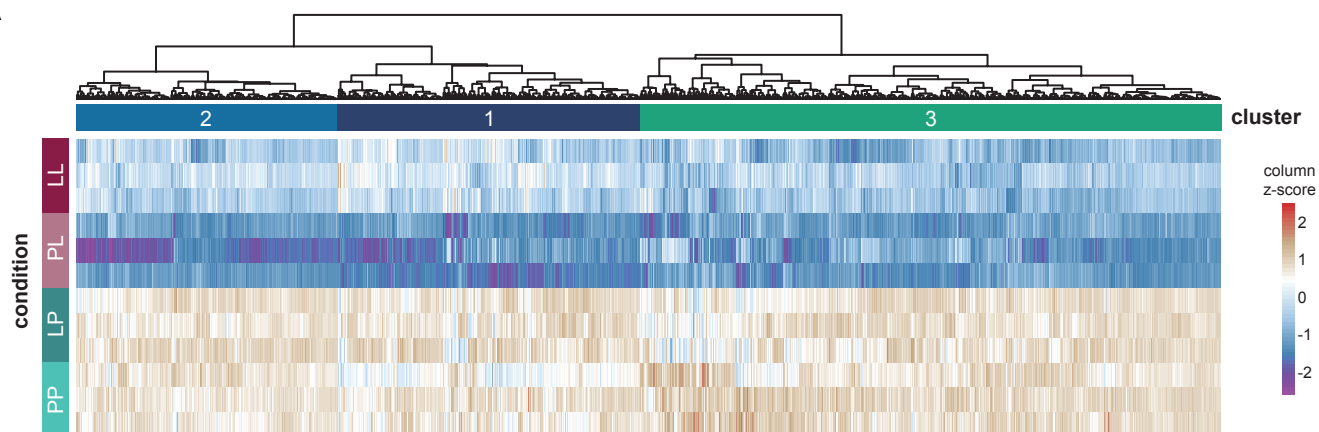

B

### top20 GO: upregulated genes by LPS

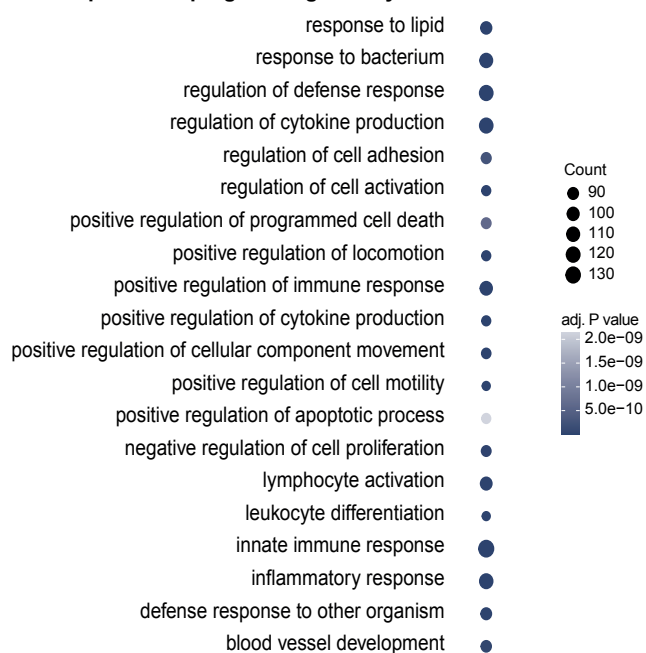

C

### top20 GO: downregulated genes by LPS

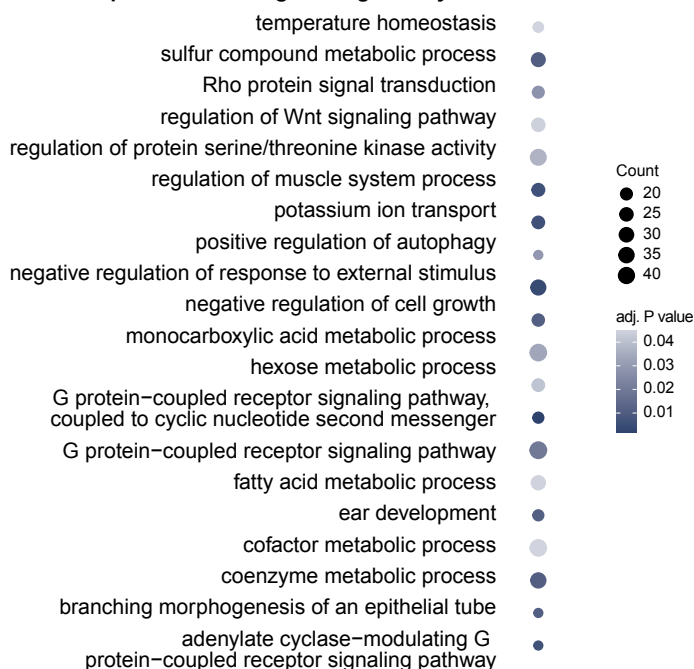

Supplement: Supplementary file 3 — Additional file 3: Fig. S3. Related to Fig. 2, LPS-downregulated genes and associated GO terms. a, Heatmap with Manhattan distance-based hierarchical clustering analysis of downregulated genes in response to LPS in microglia of C57BL/6 mice (n = 3 per experimental condition) three hours after i.p. injection with LPS (LogFC > 1 and FDR < 0.01, PP versus PL). b, c, Gene ontology (GO) analysis of genes upregulated (b) and downregulated (c) 3 h after LPS challenge in microglia of C57BL/6 mice. Based on gene count per GO term, the top 20 GO terms were identified. The size of the dot represents the gene count per GO term and the color indicates the adjusted P value. [file 12974_2022_2463_MOESM3_ESM.pdf]

A

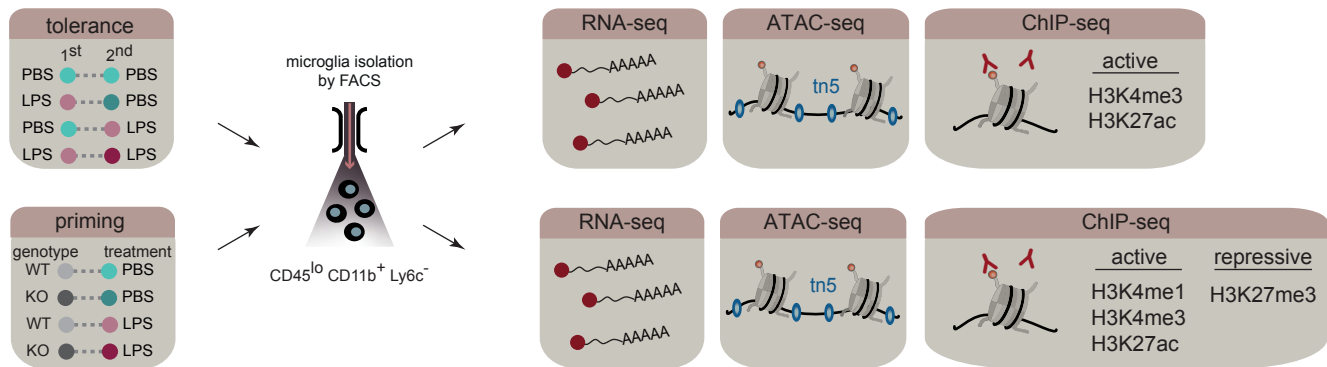

B

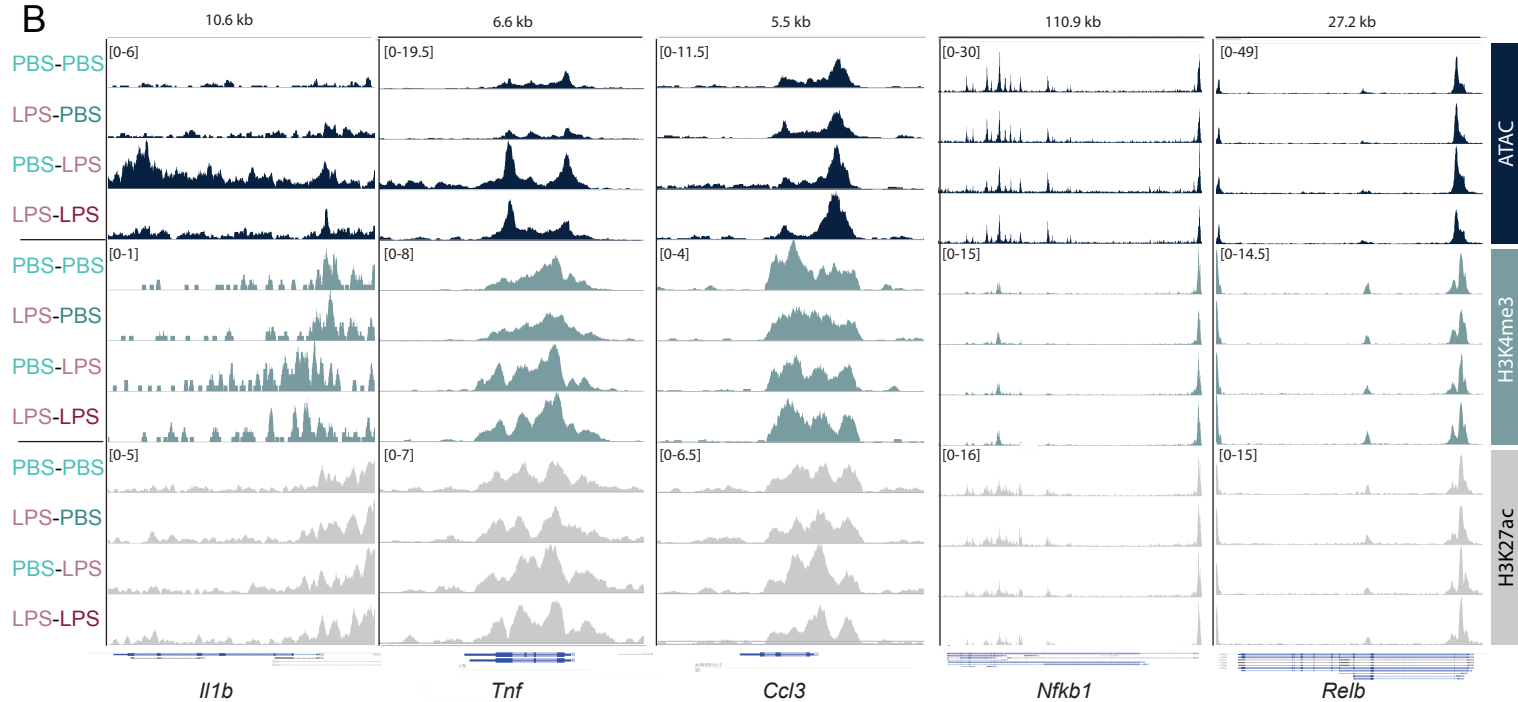

C

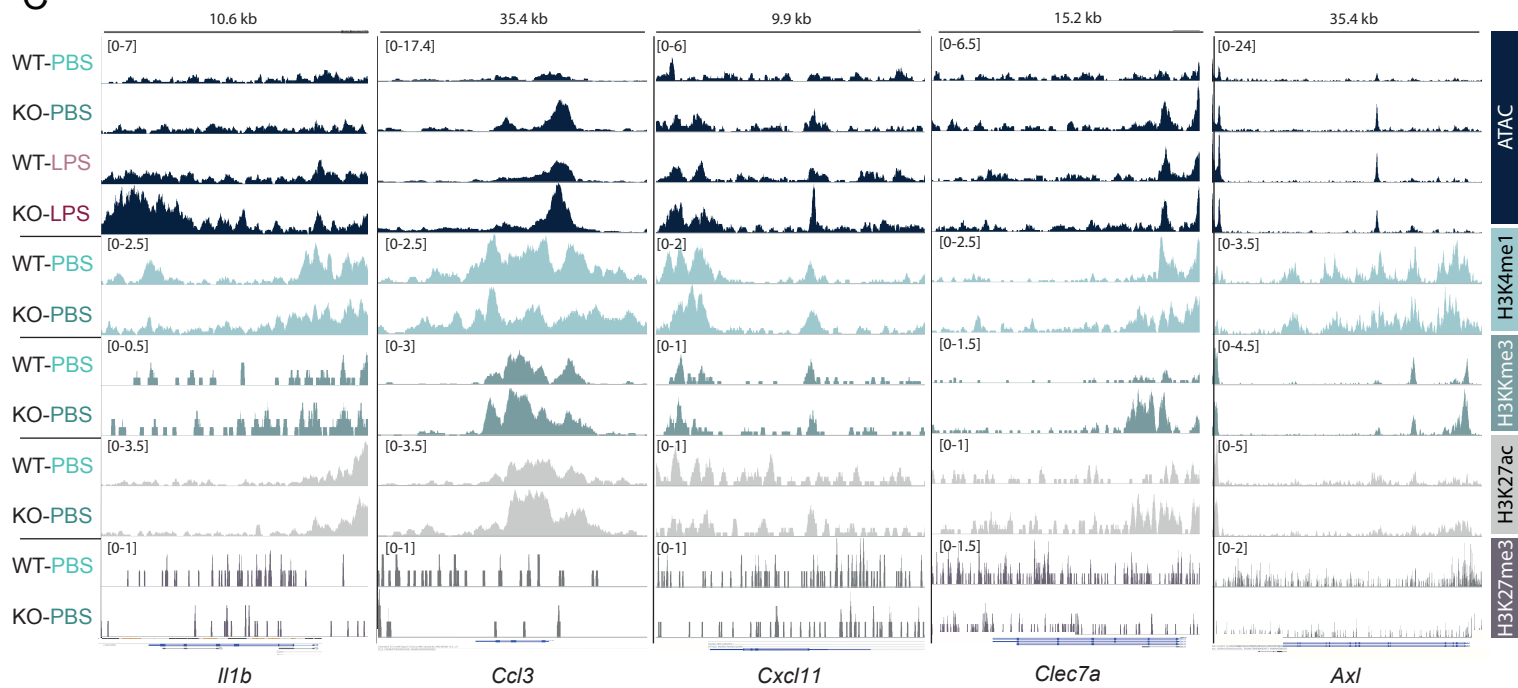

Supplement: Supplementary file 4 — Additional file 4: Fig. S4. Related to Figs. 3 and 4, ATAC- and ChIP-sequencing peak enrichment at representative desensitized and primed gene loci. a, Experimental strategy for the analysis of chromatin accessibility, and occupation by histone modifications. H3K4me3 and H3K27ac were analyzed in ‘desensitized’ and ‘tolerant’ microglia. H3K4me1, H3K4me3 and H3K27ac were determined in ‘primed’ microglia. b, c, Tracks of ATAC and indicated histone marks sequencing data of representative desensitized/tolerant (b) and primed/trained (c) genes. For ChIP, chromatin of 5 mice per experimental group was pooled; for ATAC, microglia (80,000 total) from 2 mice per experimental group were pooled. Tracks were visualized using JetBrains SPAN peak analyzer. Gene expression of these genes are shown in S2F and S2G. [file 12974_2022_2463_MOESM4_ESM.pdf]
